# Stereotactic ablative body radiotherapy with a central high dose using CyberKnife for metastatic lung tumors

**DOI:** 10.1186/s12885-023-10635-6

**Published:** 2023-03-07

**Authors:** Kazuhiko Hayashi, Osamu Suzuki, Hiroya Shiomi, Hitoshi Ono, Akira Setoguchi, Masataka Nakai, Erina Nakanishi, Shotaro Tatekawa, Naoko Ose, Takero Hirata, Keisuke Tamari, Yuji Seo, Soichiro Funaki, Fumiaki Isohashi, Shinichi Shimizu, Yasushi Shintani, Kazuhiko Ogawa

**Affiliations:** 1 Department of Radiation Oncology, Osaka University Graduate School of Medicine, 2-2 (D10) Yamada-Oka, Suita, Osaka, Japan; 2 Osaka Heavy Ion Therapy Center, Osaka, Japan; 3 Department of General Thoracic Surgery, Osaka University Graduate School of Medicine, Osaka, Japan

**Keywords:** Stereotactic ablative body radiotherapy, SBRT, CyberKnife, Metastatic lung tumor

## Abstract

**Background:**

The CyberKnife system features a robotically-positioned linear accelerator to deliver real-time image-guided stereotactic ablative body radiotherapy (SABR). It achieves steep dose gradients using irradiation from hundreds of different directions and increases the central dose of the gross tumor volume (GTV) without increasing the marginal dose to the planning target volume. We evaluated the effectiveness and safety of SABR with a central high dose using CyberKnife for metastatic lung tumors.

**Methods:**

A total of 73 patients with 112 metastatic lung tumors treated with CyberKnife were retrospectively analyzed. Local control, progression-free survival, and overall survival were calculated using the Kaplan–Meier method. The median age was 69.2 years. The most common primary sites were the uterus (*n* = 34), colorectum (*n* = 24), head and neck (*n* = 17), and esophagus (*n* = 16). For peripheral lung tumors, the median radiation dose was 52 Gy in 4 fractions, whereas for centrally located lung tumors, it was 60 Gy in 8–10 fractions. The dose prescription was defined as 99% of the solid tumor components of the GTV. The median maximum dose within the GTV was 61.0 Gy. The GTV and planning target volume were enclosed conformally by the 80% and 70% isodose lines of the maximum dose, respectively. The median follow-up period was extended to 24.7 months; it was 33.0 months for survivors.

**Results:**

The 2-year local control, progression-free survival, and overall survival rates were 89.1%, 37.1%, and 71.3%, respectively. Toxicities of grade ≥ 2 were noted as grade 2 and 3 radiation pneumonitis in one patient each. The two patients with grade 2 or higher radiation pneumonitis had both received simultaneous irradiation at two or three metastatic lung tumor sites. No toxicity of grade ≥ 2 was observed in patients with metastasis in one lung only.

**Conclusions:**

SABR with a central high dose using CyberKnife for metastatic lung tumors is effective with acceptable toxicity.

**Trial registration:**

Number: 20557, Name: Stereotactic ablative radiotherapy using CyberKnife for metastatic lung tumor, URL: http://www.radonc.med.osaka-u.ac.jp/pdf/SBRT.pdf, Date of registration: April 1, 2021 (retrospectively registered), Date of enrollment: May 1, 2014.

## Background

As a form of local therapy, surgery is the standard treatment for pulmonary oligometastases in various cancers, such as colorectal cancer [1]. For elderly patients or patients with serious comorbidities, stereotactic ablative body radiotherapy (SABR) is administered as an alternative to surgery. SABR is defined as the precise delivery of high-dose hypofractionated radiation, with little damage to the surrounding normal tissues. CyberKnife ® (Accuray, Sunnyvale, CA, USA) is a radiation device that delivers SABR. The CyberKnife system features a robotically-positioned linear accelerator that delivers real-time image-guided stereotactic radiotherapy. This occurs through synchronous respiratory tracking technology and it achieves steep dose irradiation gradients from hundreds of different directions [2].

Many studies have reported the use of the SABR delivery method using a linear accelerator or the CyberKnife for metastatic lung tumors [3–6]. However, the prescribed dose, fractionation, and treatment results varied in these studies. Concretely, the prescribed dose ranged from 23 to 60 Gy delivered in 1–10 fractions. The 2-year local control (LC) and overall survival (OS) rates ranged from 31 to 93% and from 40 to 85%, respectively. Moreover, although several studies about the use of CyberKnife for the treatment of metastatic lung tumors have been reported so far, evidence around its safety and effectiveness is still lacking [3–5]. Therefore, the SABR methodology for the CyberKnife has not yet been established.

Miura et al. in their study using patient treatment plans and phantoms reported that the gross tumor volume (GTV) prescriptions are more optimized than the planning target volume (PTV) prescriptions [7]. Furthermore, they reported that the GTV prescription guarantees a stable dose to GTV during respiratory movements [8]. These results led us to using GTV dose prescription. In addition, with the evolution of radiotherapy equipment, such as the development of a linear accelerator, or CyberKnife, it has become possible to increase the central dose of GTV while keeping the marginal dose of PTV unaltered. This method has the potential to increase LC of metastatic lung tumors without increasing toxicity for the normal tissues surrounding the PTV. In fact, Takeda et al. were the first to report that SABR with a central high dose using a linear accelerator achieved a high LC and was safe for metastatic lung tumors and primary lung cancers [9–11]. However, previous studies about this method have never reported data on its safety and effectiveness. In this study, we evaluated the outcomes of 73 patients (112 tumors) with metastatic lung tumors who received SABR, at a central high dose, using CyberKnife at Osaka University Hospital.

## Methods

### Patients

This study was approved by our institutional review board (approval number: 20557). Research was conducted in accordance with the Helsinki Declaration. In this study, we retrospectively evaluated the data of 73 patients (112 tumors) with metastatic lung cancer. They received SABR using CyberKnife between October 2014 and September 2021. We treated patients with three or fewer lung metastases who had received radical treatment for their primary sites and showed no recurrence outside the lungs.

### CyberKnife treatment

All the patients were treated with CyberKnife® G4 (Accuray, Sunnyvale, CA, USA). For respiratory management, patients were administered SABR using either the fiducial-less, direct tumor tracking system (XSight Lung Tracking System®, Accuray, Sunnyvale, CA, USA) or a tracking system involving skeletal structures (XSight Spine Tracking System®, Accuray, Sunnyvale, CA, USA) without implanting fiducials.

**Fig. 1:**
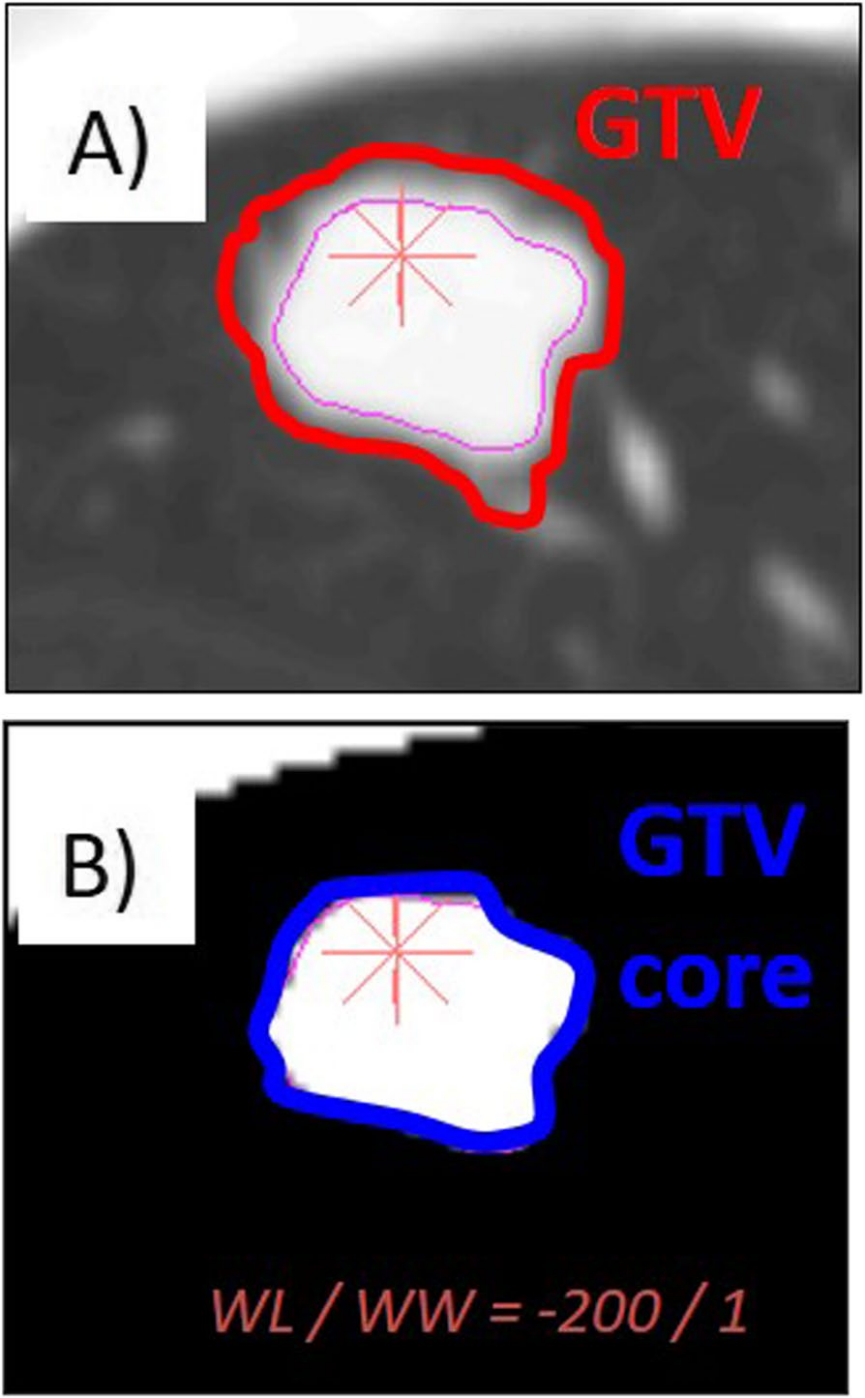
CT images of contoured GTV (**A**) and GTV core (the solid tumor components of the GTV) **B**. GTV was delineated in the lung window CT setting, while GTV core were contoured (window level [WL], –200 Hounsfield units; window width [WW], 1 Hounsfield unit). GTV, gross tumor volume

Radiotherapy was performed as per our previous report [12]. Four-dimensional computed tomography (CT), with a slice thickness of 1 mm, was performed. Primary lesions were delineated as GTV in the lung window CT setting. On the CT images, the solid tumor components (GTV core) of the GTV (window level, –200 Hounsfield units; window width, 1 Hounsfield unit), were contoured (Fig. 1). The internal target volume (ITV) was calculated from the GTVs during each respiratory phase. Finally, PTV was set as the ITV, plus a 3–8-mm safety margin. The PTV margin depends on the tracking system used, and when we use XSight Spine Tracking System®, the distance from the vertebrae.

In cases with GTV cores, a dose prescription was defined as 99% of the GTV core. Overall, 93 peripheral lung tumors were prescribed a dose of 52 Gy in 4 fractions. Additionally, the centrally located lung tumors received 60 Gy (13 tumors) in 10 fractions, 70 Gy (1 tumor) in 10 fractions, 60 Gy (1 tumor) in 8 fractions, and 56 Gy (1 tumor) in 8 fractions. In cases where the tumor did not have a solid component and had a ground-glass shadow, the dose prescription was defined as 95% of the PTV, based on which 3 tumors were prescribed 42 Gy in 4 fractions. This translated to a biologically effective dose (BED) 10 of 86.1 Gy, which is approximately equivalent to the prescription dose of 48 Gy to the isocenter. These values were reported by the Japan Clinical Oncology Group (JCOG) 0403 trial for primary lung cancer [13]. We used the Monte Carlo (Multiplan®, Accuray, Sunnyvale, CA, USA) dose calculation algorithm to determine the final doses.

### Patient follow-up visits

After the completion of treatment, patients were followed up every 3 months, for 2 years, and then every 6 months, unless there were serious complications. CT images were obtained, and positron emission tomography/CT was performed if necessary. The severity of toxicities was assessed according to the Common Terminology Criteria for Adverse Events version 4.0, published by the National Cancer Institute [14]. Information on grade ≥ 2 toxicities was collected throughout the follow-up visits.

### Statistical analyses

LC, progression-free survival (PFS), and OS were calculated using the Kaplan–Meier method. LC was defined as the duration marking the start of irradiation till the detection of tumor regrowth, within the PTV or the last follow-up. PFS was defined as the time marking the start of irradiation till the detection of disease progression at any site, death from any cause, or the last follow-up. OS was defined as the time marking the start of irradiation till death or the last follow-up. To determine the prognostic factors for LC, PFS, and OS, univariate analysis was performed using the log-rank test. The patients were divided into sub-groups according to their median age. Based on tumor diameters and BED10, the patients were divided into sub-groups according to previous reports [3, 4, 15]. Multivariate analysis was performed using the Cox proportional hazards model based on variables showing significant *P*-values in univariate analysis. A two-sided P < 0.05 was considered statistically significant. All statistical analyses were performed using JMP statistical software (version 16; SAS Institute, Cary, NC, USA).

## Results

### Patient characteristics

All 73 patients with 112 lung tumors were treated on schedule. For metachronous or synchronous metastatic lung tumors, 24 patients received two or more SABRs using CyberKnife. The median follow-up period extended to 24.7 (range, 4.0–87.7) months. The median follow-up period was 33.0 months for the survivors. Patient characteristics are summarized in Table 1. The median age of the patients was 69.2 (range, 34.0–88.6) years. The most common primary sites were the uterus (*n* = 34), colorectum (*n* = 24), head and neck (*n* = 17), and esophagus (*n* = 16). Overall, 47, 41, and 12 patients had squamous cell carcinoma; adenocarcinoma, including endometrial adenocarcinoma; and sarcoma, including carcinosarcoma, respectively. The median tumor diameter was 11.5 mm.

**Table 1:** Patient characteristics

Factors	Value or number
Age	
Median, years (range)	69.2 (34.0–88.6)
Sex	
Male	55
Female	57
PS	
0	82
1	28
2	2
Smoking status	
Current or previous smoker	52
Never	40
Unknown	20
History of irradiation to the chest	
Yes	23
No	89
Primary site	
Uterus	35
Colorectum	24
Head and neck	17
Esophagus	16
Lung	8
Pancreas	4
Breast	3
Bladder	1
Stomach	1
Liver	1
Kidney	1
Testicle	1
Histology	
SCC	47
Adenocarcinoma (including endometrial adenocarcinoma)	41
Sarcoma or carcinosarcoma	12
Spindle cell carcinoma	2
Ductal carcinoma	2
Non-small cell carcinoma	2
Transitional cell carcinoma	1
Carcinoid	1
Hepatocellular carcinoma	1
Seminoma	1
Clear cell carcinoma	1
Large cell carcinoma	1
Chemotherapy within 3 months prior to irradiation	
Yes	29
No	83
Tumor diameter	
Median (range), mm	11.5 (3.0–51.0)
Follow-up period	
Median (range), months	24.7 (4.0–87.7)
Dmax, Gy	
Median (range)	61.0 (55.1–97.2)
D99 of GTV, Gy	
Median (range)	50.1 (43.6–64.9)
D95 of PTV, Gy	
Median (range)	42.3 (39.4–55.9)
PTV volume, cm^3^	
Median (range)	11.6 (2–134.1)

*PS* performance status, *SCC* squamous cell carcinoma, *Dmax* maximum radiation dose, *D99 or D95* minimum dose that covered 99% or 95% of the target volume, respectively, *GTV* gross tumor volume, *PTV* planning target volume

The median maximum dose, within the GTV, was 61.0 Gy. The median of 99% of the GTV and 95% of the PTV were 50.1 Gy and 42.3 Gy, respectively. The GTV and PTV were conformally enclosed by 80% and 70% isodose lines of the maximum dose, respectively.

### Local control and patient survival

**Fig. 2:**
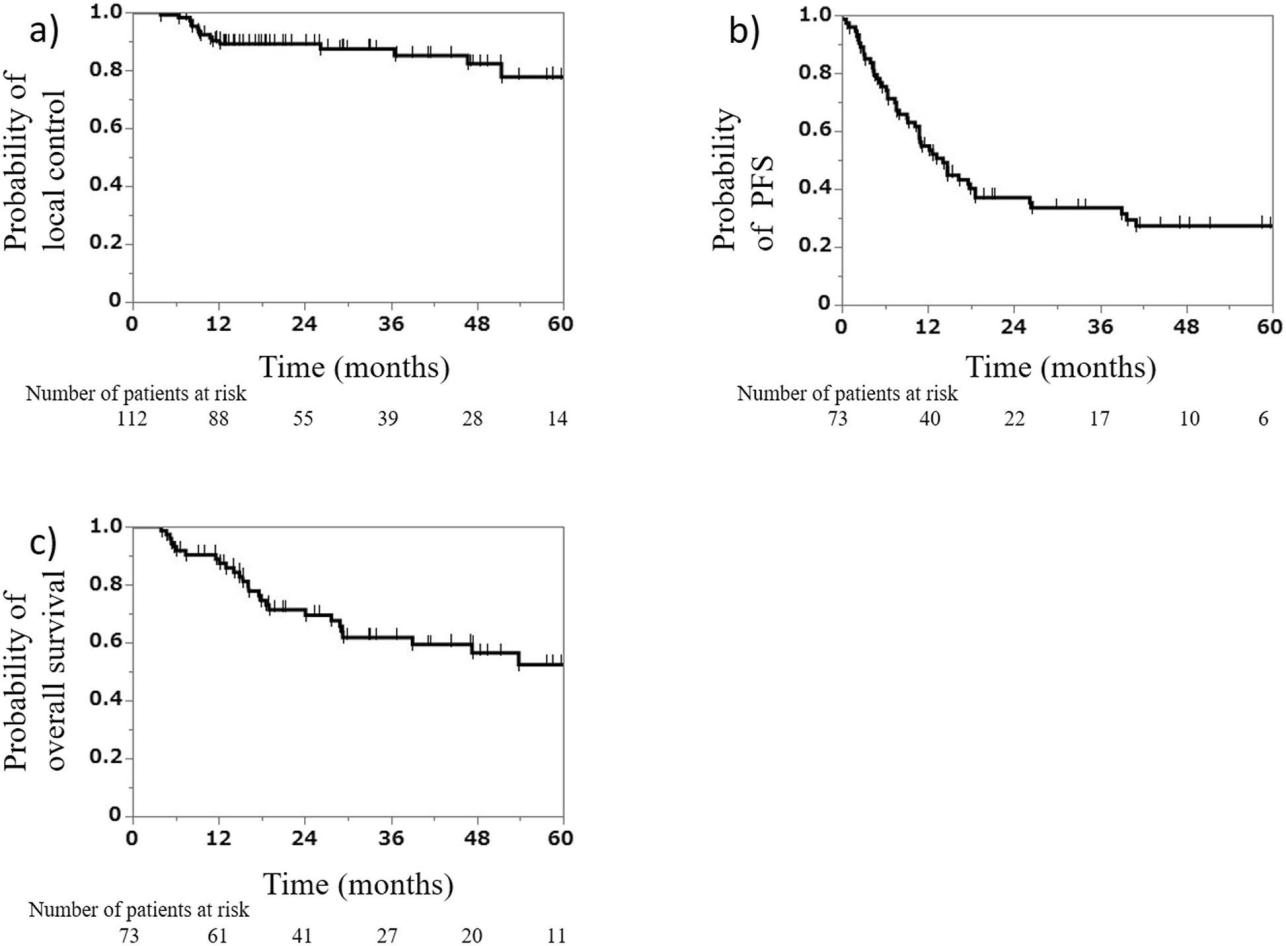
Kaplan–Meier curves. Local control (**a**) (*n* = 112 tumors), progression-free survival (**b**) (*n* = 73 patients), and overall survival (**c**) (*n* = 73 patients). PFS, progression-free survival

By the end of the follow-ups, 23 (31.5%) and 6 (8.2%) of the 73 patients had either died of cancer or due to unrelated causes, respectively. A total of 44 patients survived. During the first relapse, 7 (6.3%) of the 112 tumors demonstrated local recurrence within the PTV, 38 (33.9%) had recurrent lung tumors outside the PTV, and 39 (34.8%) had recurrence at other sites. The 2-year LC, PFS, and OS rates were 89.1% (95% confidence interval [CI], 81.4%–93.9%), 37.1% (95% CI, 26.6%–48.9%), and 71.3% (95% CI, 59.2%–80.9%), respectively (Fig. 2a–c).

### Toxicities

Toxicities of grade ≥ 2 were found to be grade 2 and 3 radiation pneumonitis in one patient (1.4%) each. The two patients with grade 2 or higher radiation pneumonitis had both received simultaneous irradiation at two or three metastatic lung tumor sites. No grade ≥ 2 toxicities were observed in patients in whom the metastasis was detected only in one lung.

### Prognostic analysis

**Fig. 3:**
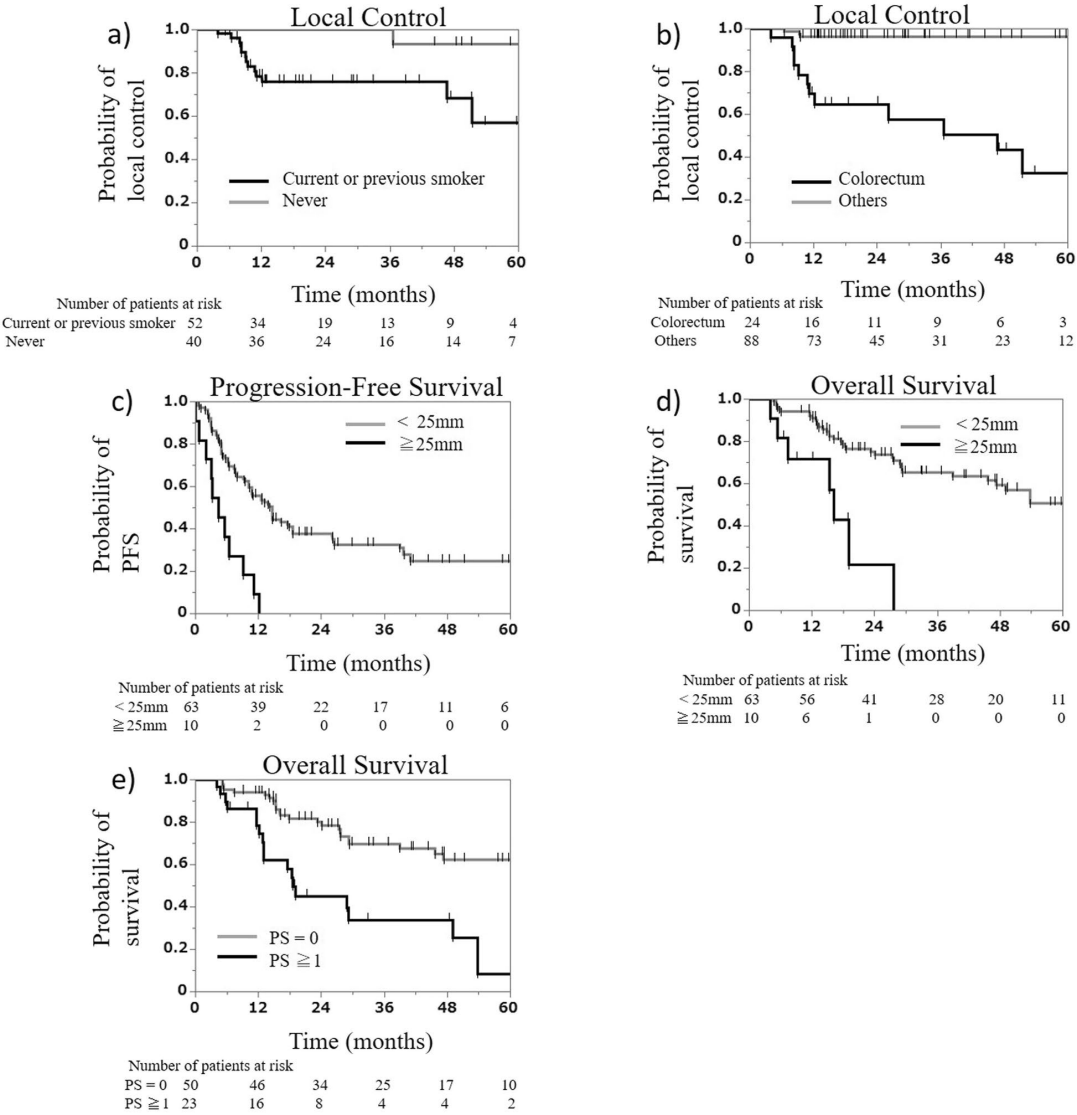
Kaplan–Meier curves. Local control rate according to smoking status (**a**) and primary site **b**. Progression-free survival rate according to tumor diameter **c**. Overall survival rate according to tumor diameter (**d**) and performance status **e**. PFS, progression-free survival; PS, performance status

Univariate analysis of prognostic factors associated with LC, PFS, and OS showed that smoking status, primary site, histology findings, and tumor diameter were significant prognostic factors for LC. Performance status (PS), smoking status, and tumor diameter were significant prognostic factors for OS (Table 2); multivariate analysis was performed using these factors. Consequently, smoking status (*P* = 0.024) and primary site (*P* = 0.012) were significant prognostic factors for LC. Furthermore, tumor diameter (*P* = 0.002) was a significant prognostic factor for PFS. Finally, PS (*P* = 0.021) and tumor diameter (*P* = 0.018) were significant prognostic factors for OS (Table 2). Additionally, the 2-year LC rates of current or previous smokers and non-smokers were 75.7% and 100%, respectively (Fig. 3a). The 2-year LC rates for colorectal cancer and others were 64.7% and 96.2%, respectively (Fig. 3b). The 2-year PFS rates of patients with tumor diameter < 25 mm and ≥ 25 mm were 37.6% and not reached, respectively (Fig. 3c). The 2-year OS rates of patients with tumor diameter < 25 mm and ≥ 25 mm were 74.9% and 21.5%, respectively (Fig. 3d). The 2-year OS rates of patients with PS = 0 and ≥ 1 were 79.9% and 45.2%, respectively (Fig. 3e).

**Table 2:** Univariate and multivariate analyses of local control, progression-free survival, and overall survival

		**Local control**				**Progression-free survival**			**Overall survival**		
		**Univariate analysis**	**Multivariate analysis**			**Univariate analysis**	**Multivariate analysis**		**Univariate analysis**	**Multivariate analysis**	
**Factors**	**No. of patients**	***P*-value**	**Hazard ratio** **(95% CI)**	***P*-value**	**No. of patients**	***P*-value**	**Hazard ratio** **(95% CI)**	***P*-value**	***P*-value**	**Hazard ratio**	***P*-value**
Age (years)		0.719				0.988			0.441		
<68.7	56				30						
≥68.7	56				43						
PS		0.708				0.008		0.187	0.002	2.62	0.021
0	82				50					(1.19-5.80)	
1–2	30				23						
Sex		0.142				0.427			0.089		
Male	55				31						
Female	57				42						
Smoking status		0.002	6.80	0.024		0.040		0.303	0.092		
Current or previous	40		(1.84-54.71)		35						
Never	52				21						
Primary site		<0.001	1.3*10^9^	0.012		0.553			0.186		
Colorectum	23		(0-NC)		16						
Others	89				57						
Histology		0.011		0.466		0.078			0.334		
SCC	47				33						
Adenocarcinoma	41				26						
Sarcoma	12				5						
Others	12				9						
Tumor diameter		<0.001		0.071		<0.001	4.23	0.002	<0.001	3.78	0.018
<25 mm	101				63		(1.77-10.1)			(1.37-10.45)	
≥25 mm	11				10						
Chemotherapy within 3 months before SABR		0.246				0.650			0.575		
Yes	29				22						
No	83				51						
Numbers of SABR		0.439				0.280			0.968		
1 location	49				49						
2 or more locations	63				24						
BED10		0.124				0.077			0.334		
<100 Gy	17				9						
≥100 Gy	95				64						

*PS* performance status, *SCC* squamous cell carcinoma, *SABR* stereotactic ablative radiotherapy, *BED10* biologically effective dose with alpha/beta=10, *NC* not calculable, *CI* confidence interval

## Discussion

We performed SABR with CyberKnife for metastatic lung tumors, which achieved steep dose gradients with irradiation from hundreds of directions. We delivered radiation to the tumors with a central high dose while keeping the marginal dose of the PTV low. The median value of the maximum dose within the GTV was 61.0 Gy and the median of 99% of the PTV was 42.3 Gy. Consequently, our results showed a high LC rate of 89.1% at 2 years and a low toxicity rate of 1.4% (grade 3 radiation pneumonitis). Our findings suggest that our new method of SABR with a central high dose using CyberKnife is a reliable option in patients with pulmonary lung metastasis. However, the dose prescriptions of SABR still vary and there is no unified method.

Some reviews have comprehensively assessed the delivery of SABR to metastatic lung tumors (Table 3). Siva et al. examined 29 reports that analyzed a total of 564 metastatic lung tumors. The study reported that the 2-year weighted LC and OS rates were 77.9% and 53.7%, respectively [16]. Virbel et al. evaluated 18 reports that studied 1705 metastatic lung tumors in total, and reported that the 2-year LC and OS rates ranged from 31 to 93% and 40% to 85%, respectively [6]. These reports were regarding metastatic lung tumors treated with a linear accelerator or CyberKnife. Several studies on SABR using CyberKnife for metastatic lung tumors showed that the 2-year LC and OS rates ranged from 71.0% to 90.6% and 61.3% to 63.0%, respectively [3, 4, 17]. Our results showed 2-year LC and OS rates of 89.1% and 71.3%, respectively. It also demonstrated that the outcomes of SABR using CyberKnife are favorable in comparison to those in previous reports.

**Table 3:** Comparison of SABR using a linear accelerator or CyberKnife for metastatic lung tumors

Author	A radiation device	No. of patients	2-year local control	2-year OS	Grade ≥ 3 toxicity
Wang et al [3].	CK	95	90.6%	61.3%	Grade 3 radiation pneumonitis, 3.2%
Sharma et al [4].	CK	206	85%	63%	Grade 3 dyspnea, 1.5%; Grade chest paint, 0.5%
Brown et al [17].	CK	35	71%		Grade 3–4 pulmonary toxicity, 2.8%
Siva et al [16]. A systematic review	Liniac and CK	334	77.9%	53.7%	Grade ≥ 3 toxicity 4%
Virbel et al [6]. A systematic review	Liniac and CK	1191	31–93%	40–85%	NA
*Present study*	CK	73	89.1%	71.3%	Grade 3 radiation pneumonitis, 1.4%

*CK* CyberKnife, *NA* not applicable, *OS* overall survival. Number of cases of toxicities noted, but no percentage noted

Siva et al. performed a systematic review of toxicities following SABR for metastatic lung tumors and reported that the incidence rate of grade ≥ 3 toxicities, including radiation pneumonitis, was 4% [16]. In some studies on SABR using CyberKnife, grade ≥ 3 radiation pneumonitis was detected in 2.0–3.2% of patients [3, 4]. The present study revealed that grade 3 radiation pneumonitis was observed in 1 (1.4%) patient, and grade ≥ 4 toxicity was not observed. These results indicate that the incidence of grade ≥ 3 toxicities is slightly lower than that reported in previous studies.

The dose prescribed for SABR in metastatic lung tumors varies. A previous study showed that the dose prescription to the GTV is more optimized than that to the PTV [16, 17]. Therefore, we adopted these dose prescriptions to the GTV core in our study. Of note, our dose prescription was defined as 52 Gy to 99% of the GTV core. In addition, the CyberKnife could achieve steep dose gradients with the median value of maximum dose within GTV of 61.0 Gy and 95% of the PTV of 42.3 Gy. This indicates that the PTV is enclosed conformally by the 70% isodose line of the maximum dose, approximately. The dose of 42 Gy for 95% of the PTV was based on the JCOG 0403 trial, which translates to a BED10 of 86.1 Gy [13]. According to several studies that excluded single-fractionated irradiation, the dose prescription was 45–60 Gy in 3–5 fractions to 95% of the PTV. Furthermore, the prescription isodoses varied from 70 to 95%, and BED10 < 100 Gy of the PTV was a significantly poor predictor of LC [3–5, 16, 18]. Our dose to the PTV was approximately equivalent to 87 Gy at BED10, which is a lower dose than previously reported. Therefore, it is expected to result in poorer LC. However, as mentioned above, our study demonstrated that LC fared favorably over the previous reports owing to the low toxicity rate. This may be due to SABR with a central high dose using CyberKnife. In other words, our irradiation method of increasing the central dose of the GTV, while decreasing the peripheral dose of the PTV, may be suitable for SABR of metastatic lung tumors.

Based on Garcia et al.’s. report indicating that tumor diameter > 25 mm was a prognostic factor, we set 25 mm as the cutoff value. In the multivariate analysis, we found that the smoking status and primary site, tumor diameter, or PS and tumor diameter were significant prognostic factors for LC, PFS, or OS, respectively. The prognostic factors of primary site and tumor diameter were consistent with those stated in previous reports [15, 18, 19]. We speculate that the reason for the poor prognosis of patients with lesser PS is owing to difficulties in administering systemic chemotherapy. Additionally, it is unclear why smoking status influences LC.

Our study has four limitations. First, it was a single-center retrospective analysis. Second, a group of patients who had difficulty or refused surgery was included leading to a patient bias. Third, our study used GTV prescription instead of the common PTV prescription, which may have led to unexpected bias. Fourth, the total dose, fractionation schedule, tumor sites (peripheral or central), and primary sites varied. Therefore, further large-scale, multicenter, prospective trials are warranted.

## Conclusions

We irradiated tumors using the CyberKnife with a central high dose while keeping the marginal dose of the PTV low. SABR with a central high dose using CyberKnife for metastatic lung tumors is effective and has an acceptable toxicity level. Furthermore, it may be administered as an alternative to surgery for elderly patients or patients with serious comorbidities.

## Data Availability

The datasets used and/or analyzed during the current study are available from the corresponding author on reasonable request.

## Abbreviations

SABR: Stereotactic ablative body radiotherapy
GTV: Gross tumor volume
PTV: Planning target volume
LC: Local control
OS: Overall survival
GTV core: Solid tumor components of the GTV
ITV: Internal target volume
BED: Biologically effective dose
JCOG: Japan Clinical Oncology Group
PFS: Progression-free survival
PS: Performance status
CT: Computed tomography
CTV: Clinical target volume
CI: Confidence interval

## References

[1] Benson AIB. National Comprehensive Cancer Network. Rectal Cancer. In: Clinical Practice Guidelines in Oncology. 2017. https://www2.tri-kobe.org/nccn/guideline/colorectal/english/rectal.pdf. Accessed 30 Oct 2022.

[2] Gibbs IC, Loo BW (2010). CyberKnife stereotactic ablative radiotherapy for lung tumors. Technol Cancer Res Treat.

[3] Wang Z, Kong QT, Li J, Wu XH, Li B, Shen ZT (2015). Clinical outcomes of cyberknife stereotactic radiosurgery for lung metastases. J Thorac Dis.

[4] Sharma A, Duijm M, Oomen-de Hoop E, Aerts JG, Verhoef C, Hoogeman M (2018). Factors affecting local control of pulmonary oligometastases treated with stereotactic body radiotherapy. Acta Oncol.

[5] Fumagalli I, Bibault JE, Dewas S, Kramar A, Mirabel X, Prevost B (2012). A single-institution study of stereotactic body radiotherapy for patients with unresectable visceral pulmonary or hepatic oligometastases. Radiat Oncol.

[6] Virbel G, Le Fèvre C, Noël G, Antoni D (2021). Stereotactic body radiotherapy for patients with lung oligometastatic disease: a five-year systematic review. Cancers.

[7] Miura H, Masai N, Oh RJ, Shiomi H, Yamada K, Sasaki J (2014). Clinical introduction of Monte Carlo treatment planning for lung stereotactic body radiotherapy. J Appl Clin Med Phys.

[8] Miura H, Masai N, Oh RJ, Shiomi H, Sasaki J, Inoue T (2013). Approach to dose definition to the gross tumor volume for lung cancer with respiratory tumor motion. J Radiat Res.

[9] Tsurugai Y, Takeda A, Sanuki N, Eriguchi T, Aoki Y, Oku Y (2019). Stereotactic body radiotherapy for patients with non-small-cell lung cancer using RapidArc delivery and a steep dose gradient: prescription of 60% isodose line of maximum dose fitting to the planning target volume. J Radiat Res.

[10] Tateishi Y, Takeda A, Horita N, Tsurugai Y, Eriguchi T, Kibe Y (2021). Stereotactic body radiation therapy with a high maximum dose improves local control, cancer-specific death, and overall survival in peripheral early-stage non-small cell lung cancer. Int J Radiat Oncol Biol Phys.

[11] Takeda A, Sanuki N, Tsurugai Y, Oku Y, Aoki Y (2016). Stereotactic body radiotherapy for patients with oligometastases from colorectal cancer: risk-adapted dose prescription with a maximum dose of 83–100 Gy in five fractions. J Radiat Res.

[12] Hayashi K, Suzuki O, Shiomi H, Nakai M, Fujiwara K, Nakanishi E (2022). Stereotactic ablative radiotherapy using CyberKnife for Stage I non-small-cell lung cancer: a retrospective analysis. Anticancer Res.

[13] Kawahara D, Ozawa S, Kimura T, Saito A, Nishio T, Nakashima T (2017). Marginal prescription equivalent to the isocenter prescription in lung stereotactic body radiotherapy: preliminary study for Japan Clinical Oncology Group trial (JCOG1408). J Radiat Res.

[14] NIH Publication. Common terminology criteria for adverse events v4.03. Department of Health and Human Services. 2009. https://evs.nci.nih.gov/ftp1/CTCAE/CTCAE_4.03_2010-06-14_%0AQuickReference_5x7.pdf. Accessed 30 Oct 2022.

[15] García-Cabezas S, Bueno C, Rivin E, Roldán JM, Palacios-Eito A (2015). Lung metastases in oligometastatic patients: outcome with stereotactic body radiation therapy (SBRT). Clin Transl Oncol.

[16] Siva S, MacManus M, Ball D (2010). Stereotactic radiotherapy for pulmonary oligometastases: a systematic review. J Thorac Oncol.

[17] Brown WT, Wu X, Fowler JF, García S, Fayad F, Amendola BE (2008). Lung metastases treated by CyberKnife image-guided robotic stereotactic radiosurgery at 41 months. South Med J.

[18] Osti MF, Agolli L, Valeriani M, Reverberi C, Bracci S, Marinelli L (2018). 30-Gy single dose stereotactic body radiation therapy (SBRT): Reportreport on outcome in a large series of patients with lung oligometastatic disease. Lung Cancer.

[19] Jingu K, Matsushita H, Yamamoto T, Umezawa R, Ishikawa Y, Takahashi N (2018). Stereotactic radiotherapy for pulmonary oligometastases from colorectal cancer: a systematic review and meta-analysis. Technol Cancer Res Treat.

